# Single brain metastases – prognostic factors and impact of residual tumor burden on overall survival

**DOI:** 10.3389/fonc.2024.1330492

**Published:** 2024-03-15

**Authors:** Lea Baumgart, Aida Anetsberger, Amir Kaywan Aftahy, Benedikt Wiestler, Denise Bernhardt, Stephanie E. Combs, Hanno S. Meyer, Gerhard Schneider, Bernhard Meyer, Jens Gempt

**Affiliations:** ^1^ Department of Neurosurgery, University Medical Center Hamburg-Eppendorf, Hamburg, Germany; ^2^ Department of Neurosurgery, Klinikum rechts der Isar, School of Medicine, Technical University Munich, Munich, Germany; ^3^ Faculty of Interdisciplinary Studies, University of Applied Sciences, Landshut, Germany; ^4^ Department of Anesthesiology, Klinikum rechts der Isar, School of Medicine, Technical University Munich, Munich, Germany; ^5^ Department of Neuroradiology, Klinikum rechts der Isar, School of Medicine, Technical University Munich, Munich, Germany; ^6^ Department of Radiation Oncology, Klinikum rechts der Isar, School of Medicine, Technical University Munich, Munich, Germany; ^7^ German Cancer Consortium (DKTK), Partner Site Munich, Munich, Germany; ^8^ Institute of Innovative Radiotherapy (iRT), Department of Radiation Sciences (DRS) Helmholtz Zentrum Munich, Munich, Germany

**Keywords:** single brain metastasis, neurooncology, postoperative tumor volume, brain metastasis, postoperative MRI, extent of resection, overall survival

## Abstract

**Background:**

Brain metastases (BM) are a common and challenging issue, with their incidence on the rise due to advancements in systemic therapies and increased patient survival. Most patients present with single BM, some of them without any further extracranial metastasis (i.e., solitary BM). The significance of postoperative intracranial tumor volume in the treatment of singular and solitary BM is still debated.

**Objective:**

This study aimed to determine the impact of resection and postoperative tumor burden on overall survival (OS) in patients with single BM.

**Methods:**

Patients with surgically treated single BM between 04/2007-01/2020 were retrospectively included. Residual tumor burden (RTB) was determined by manual segmentation of early postoperative brain MRI (72 h). Survival analyses were performed using Kaplan-Meier estimates for univariate analysis and Cox regression proportional hazards model for multivariate analysis, using preoperative Karnofsky performance status scale (KPSS), age, sex, RTB, incomplete resection and singular/solitary BM as covariates.

**Results:**

340 patients were included, median age 64 years (54-71). 119 patients (35%) had solitary BM, 221 (65%) singular BM. Complete resection (RTB=0) was achieved in 73%, median preoperative tumor burden was 11.2 cm3 (5-25), and RTB 0 cm3 (0-0.2). Median OS of patients with singular BM was 13 months (4-33) vs 20 months (5-92) for solitary BM; p=0.062. Multivariate analysis revealed singular BM as independent risk factor for poorer OS: HR 1.840 (1.202-2.817), p=0.005. Complete vs. incomplete resection showed no significant OS difference (13 vs. 13 months, p=0.737). When focusing on solitary BM, complete resection led to a longer OS than incomplete resection (21 vs. 8 months), without statistical significance(p=0.250). Achieving RTB=0 resulted in higher OS for patients with solitary BM compared to singular BM (21 vs. 12 months, p=0.027). Patients who received postoperative radiotherapy (RT) had significantly longer OS compared to those without it (14 vs. 4 months, p<0.001), with favorable OS in those receiving stereotactic radiosurgery (SRS) (15 months (3-42), p<0.001) or hypofractionated stereotactic radiotherapy (HSRT).

**Conclusion:**

When complete intracranial tumor resection RTB=0 is achieved, patients with solitary BM have a favorable outcome compared to singular BM. Singular BM was confirmed as independent risk factor. There is a strong presumption that complete resection leads to an improved oncological prognosis. Patients with solitary BM tend to benefit with a favorable outcome following complete resection. Hence, surgical resection should be considered as a treatment option for patients presenting with either no or minimal extracranial disease. Furthermore, the highly favorable impact of postoperative RT on OS was demonstrated and confirmed, especially with SRS or HSRT.

## 1 Introduction

Brain metastases (BM) represent the most frequent intracranial tumors. They occur 3-10 times more often than newly diagnosed primary malignant brain tumors and represent more than 30% of intracranial tumors (1, 2). Most patients present with a single BM. Due to tremendous improvements in systemic therapy, the incidence of patients with BM is constantly increasing, as the patients have prolongated overall survival and therefore BM have more time to grow (3). Many therapy options do not penetrate the central nervous system and therefore allow brain metastases to develop. Furthermore, the extended use of neuroimaging results in an increased detection and diagnosis of BM (1).

Historically, the prognosis for patients with metastatic cancer in the brain has been poor with a median overall survival of 3-4 months (4, 5). In recent decades, studies have investigated prognostic factors that significantly improve overall survival and quality of life in these patients. In addition to age, Karnofsky performance status scale (KPSS), and spread of systematic disease, the number of BM has also been shown to be an important prognostic factor (3–6). Patients with a single BM have longer overall survival and higher quality of life (6, 7).

Single BM are divided in two subgroups: solitary and singular metastases. Singular metastasis describes the presence of a single metastasis in one organ with the simultaneous presence of other metastases in the rest of the organism.

A solitary metastasis, on the other hand, is a single metastasis with no other metastases in the same organ or in the rest of the organism.

As patients with single BM represent the largest subgroup of patients with intracranial metastases and since due to modern systemic therapies, patients are now living longer, it is crucial to analyze this cohort separately regarding prognostic factors in order to find the optimal treatment approach for this subgroup. In contrast to the surgical treatment of gliomas, where maximal cytoreduction has been established as a decisive prognostic marker, the significance of postoperative intracranial tumor volume in the treatment of single BM is still debated (8, 9). Furthermore, even though the accuracy of early postoperative MRI is significantly higher compared to intraoperative estimates, it is still not established in the neurooncological workflow (10–13). In this study, we retrospectively examined a series of 340 patients with single BM who underwent microsurgical tumor resection to determine prognostic factors and the impact of postoperative tumor burden, controlled with early postoperative MRI, on overall patient survival.

## 2 Materials and methods

### 2.1 Ethical declaration

Our study was approved by the local ethics committee (no. 5626:12). It was conducted in accordance with the ethical standards of the 1964 Declaration of Helsinki. The ethics committee decided not to demand written informed consent.

### 2.2 Patient population

We conducted a retrospective chart of patients with single BM.

Patients with a histopathological diagnosis of a single BM, pre- and postoperative MRI (within 72 hours after surgery), and tumor resection beyond biopsy met the inclusion criteria. Major demographic factors were assessed in relation to the targeted BM, including age at diagnosis, sex, smoking status, extracranial tumor activity (singular/solitary metastasis), tumor localization, pre- and postoperative KPSS, and pre- and postoperative tumor volume, were evaluated. The structure of adjuvant radiotherapy/chemotherapy as well as the date of death or the last contact were also examined. The primary endpoint was the postoperative neurological outcome. Overall survival (OS), progression-free survival (PFS), and quality of life (QoL) were secondary endpoints. For local in brain tumor control, the distant brain failure (DBF) rate was analyzed.

### 2.3 Surgery

The interdisciplinary neuro-oncology panel’s conclusions served as the basis for the decision for surgical treatment. The indication was primarily based on the following factors: 1) symptomatic lesion, 2) mass effect, 3) intratumoral bleeding, 4) ambiguous diagnosis, and 5) large posterior fossa tumors with subsequent danger of herniation/hydrocephalus. In all cases, intraoperative frozen sections were collected.

The aim of surgery was maximal cytoreduction while sparing eloquent regions of the brain. Surgeries were performed, using intraoperative neuronavigation. Furthermore, neuromonitoring and preoperative mapping were also applied, if necessary.

### 2.4 Residual tumor burden

Within 72 hours after surgery, all postoperative MRIs were examined, and postoperative tumor volume was calculated. We investigated at T1-weighted MRI sequences with and without gadolinium contrast media. As postoperative remains, any debatable reactive or contrast agent-active barrier abnormalities were classified. Complete resection was defined as residual tumor burden of 0 in the brain.

An experienced neuroradiologist (BW, 10 years of experience) and neurosurgeon (KA, 6 years of experience) performed volumetric measurements. Volumes of the contrast-enhancing tumor part were manually segmented using the Origin^®^software (Origin^®^, Brainlab, version 3.1, Brainlab AG, Munich, Germany).

### 2.5 Postoperative treatment structure and KPSS

To classify and quantify the pre- and postoperative functional status of the patient, the KPSS was used. The functional status was rated on a numerical scale ranging from 0-100, representing the patient’s ability to conduct normal activity, to undertake active work, and need for assistance with 100 representing full activity and 0 representing death.

Adjuvant therapy was selected on an individual basis for each patient after histological diagnosis by an interdisciplinary tumor board. Adjuvant radiation recommendations were based on a variety of factors, including the extent of resection (EOR), extent of disease, and the KPSS.

The institutional outpatient clinic’s electronic patient records and paper-based documentation from the treating oncologists were used to generate follow-up data.

### 2.6 Statistical analysis

Data that have a normal distribution are expressed as mean ± standard deviation (SD), non-normally distributed data as median and interquartile range (IQR).

Logistic regression analyses were performed to identify possible risk factors for outcome changes. Survival analyses were performed using Kaplan-Meier estimates for the univariate analysis. Multivariate analysis was conducted using Cox regression proportional hazards model with preoperative Karnofsky performance status scale (KPSS), age, sex, preoperative tumor volume, RTB, incomplete resection and singular/solitary BM as covariates. Additionally, when only patients with a complete resection were considered, covariates for the model were selected based on biological plausibility and significant differences between the two groups in the univariate analysis resulting in: sex, histology groups, post-op chemotherapy, post-op radiotherapy and singular/solitary BM as potential confounders considered in the Cox regression.

A two-tailed significance level of p<0.05 was defined statistically significant.

## 3 Results

### 3.1 Patient population

Between April 2007 and January 2020, the department surgically treated 362 patients with newly diagnosed single BM. A total of 340 patients met the inclusion criteria. Twenty-two patients were excluded as they underwent a biopsy only or did not receive postoperative MRI. Preoperatively, there were no significant differences between the two groups (**Table 1**). Median age at surgery was 64 years (IQR 53-71 years) and 53% of the patients were male. The cohort presented with a median preoperative KPSS of 80% (IQR 70-90%) and a median postoperative KPSS of 80% (IQR 70-90%). Median ECOG score ad admission was 1 for both groups. One hundred nineteen patients (35%) had a solitary BM, 221 patients (65%) had a singular BM (**Table 1**).

**Table 1:** Baseline characteristics of patients, tumor data and postoperative therapy.

Demographics N (%) or median (range/IQR)	All patients (n = 340)	Solitary Metastasis N = 119	Singular Metastasis N = 221	p
Sex	F 159/340 (47) M 181/340 (53)	F 61/119 (58) M 58/119 (49)	F 98/221 (44) M 123/221 (56)	0.223
Age	64 (IQR 54-71)	65 (IQR 56-73)	64 (IQR 53-71)	0.087
Karnofsky Performance Status Scale (KPSS)				
Preoperative KPSS	80% (IQR70-90)	80% (IQR70-90)	80% (IQR70-90)	0.251
Postoperative KPSS	80% (IQR70-90)	70% (IQR70-90)	80% (IQR70-90)	0.832
**Postoperative radiotherapy N (%)**				0.527
none	84/317 (24)	27/109 (25)	57/208 (27)	
WBRT	75/317(24)	30/109 (28)	45/208 (22)	
SRS	7/317 (2)	1/109 (1)	6/208 (3)	
HSRS	151/317 (48)	51/109 (47)	100/208 (48)	
**Post-op chemo**	150/340 (44)	43/119 (36)	107/221 (48)	**0.032**
**Post-op Immunotherapy**	39/289 (13)	5/95 (5)	34/194 (8)	**0.004**
Tumor burden (cm^3^) median (IQR)				
Preoperative	11.2 cm3 (5-25 cm3)	11.7 cm3 (5.2-26.8 cm3)	10.6 cm3 (4.8-24.3 cm3)	0.461
Postoperative	0 cm3 (0- 0.2 cm3)	0 cm3 (0- 0.03 cm3)	0 cm3 (0- 0.3 cm3)	0.541
**Complete resection**	247/340 (73)	89/119 (75)	158/221 (72)	0.515

Bold values mean a two-tailed significance level of p<0.05 was defined statistically significant.

Complete resection was achieved in 247/340 (73%) patients, median preoperative tumor burden was 11.2 cm3 (IQR 5-25 cm3), and median RTB was 0 cm3 (IQR 0- 0.2 cm3), with no differences between the groups. Primary tumor was most commonly lung cancer (26.2%), followed by breast (16.5%) and melanoma (14.1%). (**Table 2**)

**Table 2:** Tumor data of all patients.

Primary tumor N (%)	
NSCLC	83 (24.4.)
SCLC	6 (1.8)
Breast	56 (16.5)
Melanoma	48 (14.1)
Colon/Rectum	25 (7.2)
Carcinoma of unknown primary	23 (6.7)
Renal cell carcinoma	21 (6.2)
Stomach/Esophagus	11 (3.3)
Gynecologic	11 (3.3)
Prostate	10 (2.9)
Seminoma	5 (1.5)
Thyroid	3 (0.9)
Hepatocellular carcinoma	3 (0.9)
Other	35 (10.3)

### 3.2 Systemic disease response and local in brain tumor control

Regarding the systemic disease response, all patients with solitary brain metastases had a stable systemic disease, despite the BM. For the patients with singular BM 68/220 (31%) presented with a stable systemic disease and 152/220 (69%) had a progressive disease. For local in brain tumor control, the DBF was analyzed. After 6 months, 124 patients received an MRI for in brain tumor control. When analyzing both groups there was a distant progress reported for 46 patients after 6 months. Therefore, DBF was recorded in 5/30 (17%) (solitary) and 41/94 (44%) (singular metastases) with a significant difference between the groups (p= 0.008).

### 3.3 Survival analysis and the impact of RTB

**Figure 1:**
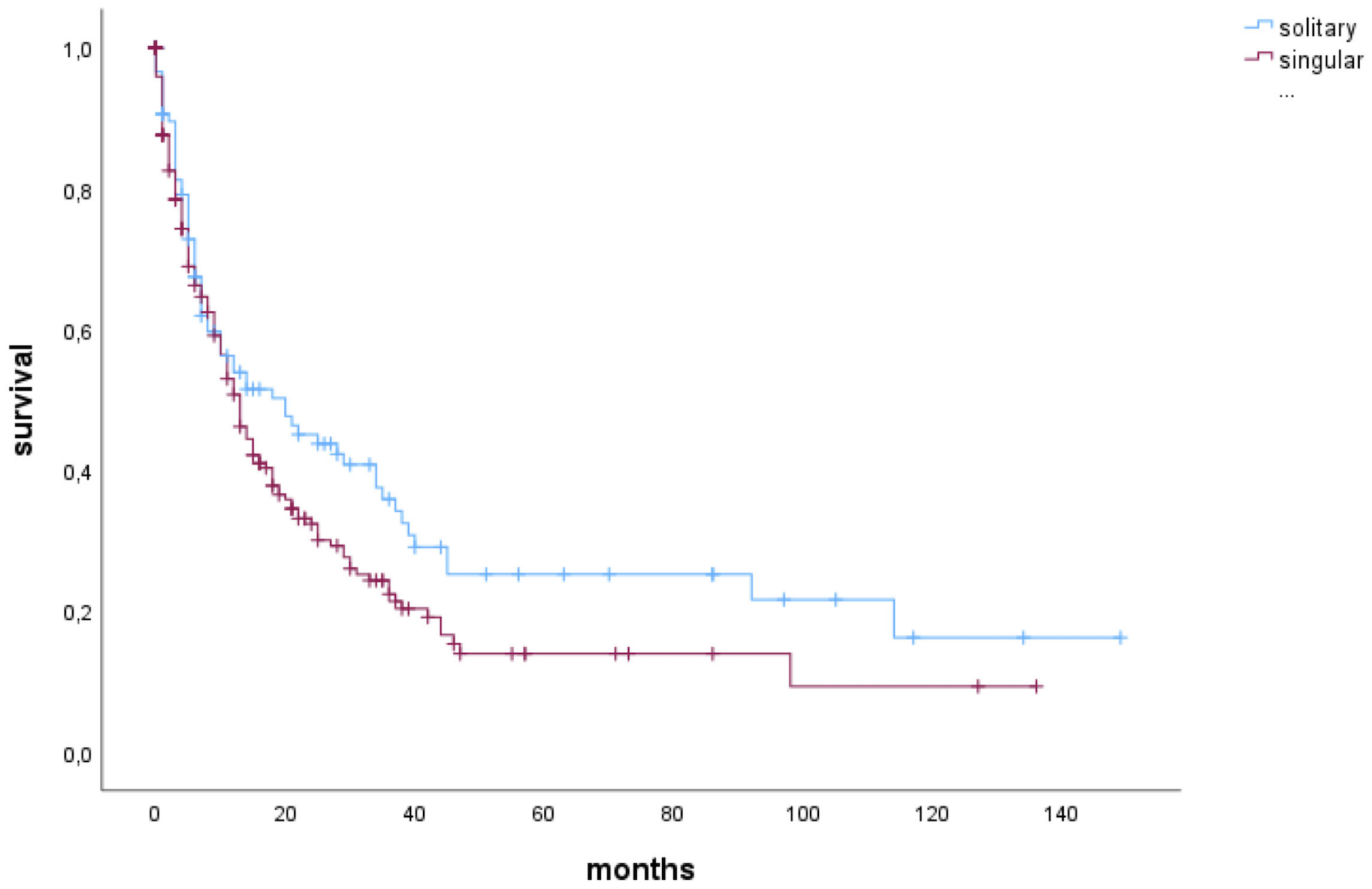
Median overall survival of patients with singular or solitary brain metastases.

Median OS of patients with singular BM was 13 months (4-33 months) and of patients with solitary BM 20 months (5-92 months; p=0.062) (**Figure 1**).

**Figure 2:**
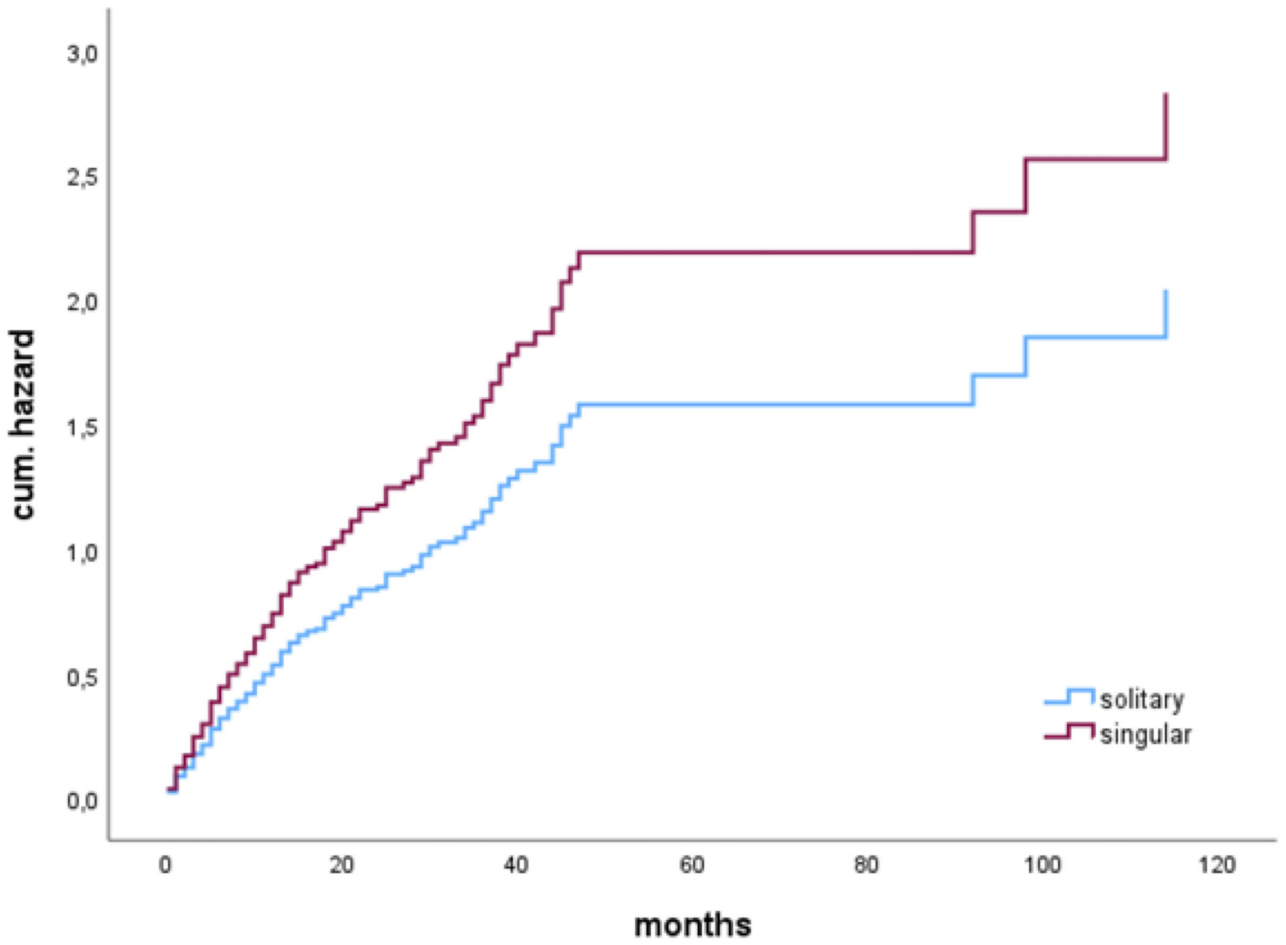
Cox proportional hazards model for singular BM (vs. solitary) as risk factor for shorter OS (HR 1.386 (1.024-1.875), p=0.034).

In patients who underwent complete resection, there was no significant difference in OS compared to those with incomplete resection (13 months vs. 13 months, p=0.737). When analyzing only patients with solitary BM, those who underwent complete resection exhibited a median OS of 21 months, while patients with incomplete resection had a median OS of 8 months. However, these findings did not reach statistical significance (p=0.250). Furthermore, the impact of RTB on OS was not statistically significant in either the singular BM group (p=0.233) or the solitary BM group (p=0.870).A multivariate Cox regression analysis considering preoperative KPSS, sex (female), age, incomplete resection, preoperative tumor volume, RTB, and singular metastasis identified singular brain metastasis to be a significant risk factor for shorter OS (HR 1.386 (1.024-1.875), p=0.034) (**Figure 2**).

**Figure 3:**
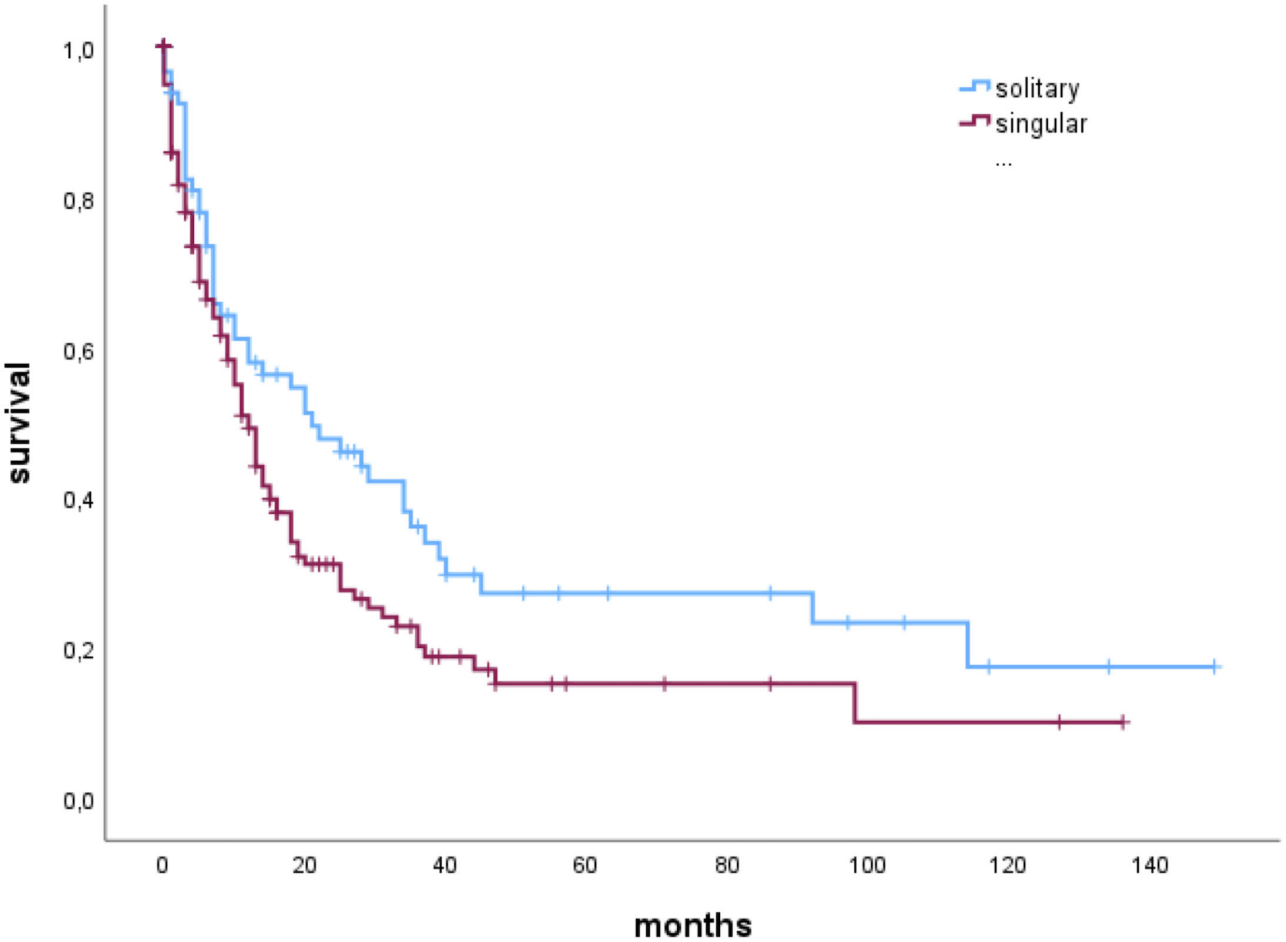
Median overall survival of patients with singular or solitary brain metastases who had a complete resection of the brain metastasis.

In a subgroup analysis in patients with a complete resection (N=247), median OS of patients with solitary BM was significantly higher than OS of patients with singular BM; solitary 21 (6-92 months), vs. singular 12 (4-31) months), as shown by Kaplan-Meier estimates (p=0.027) (**Figure 3**).

**Figure 4:**
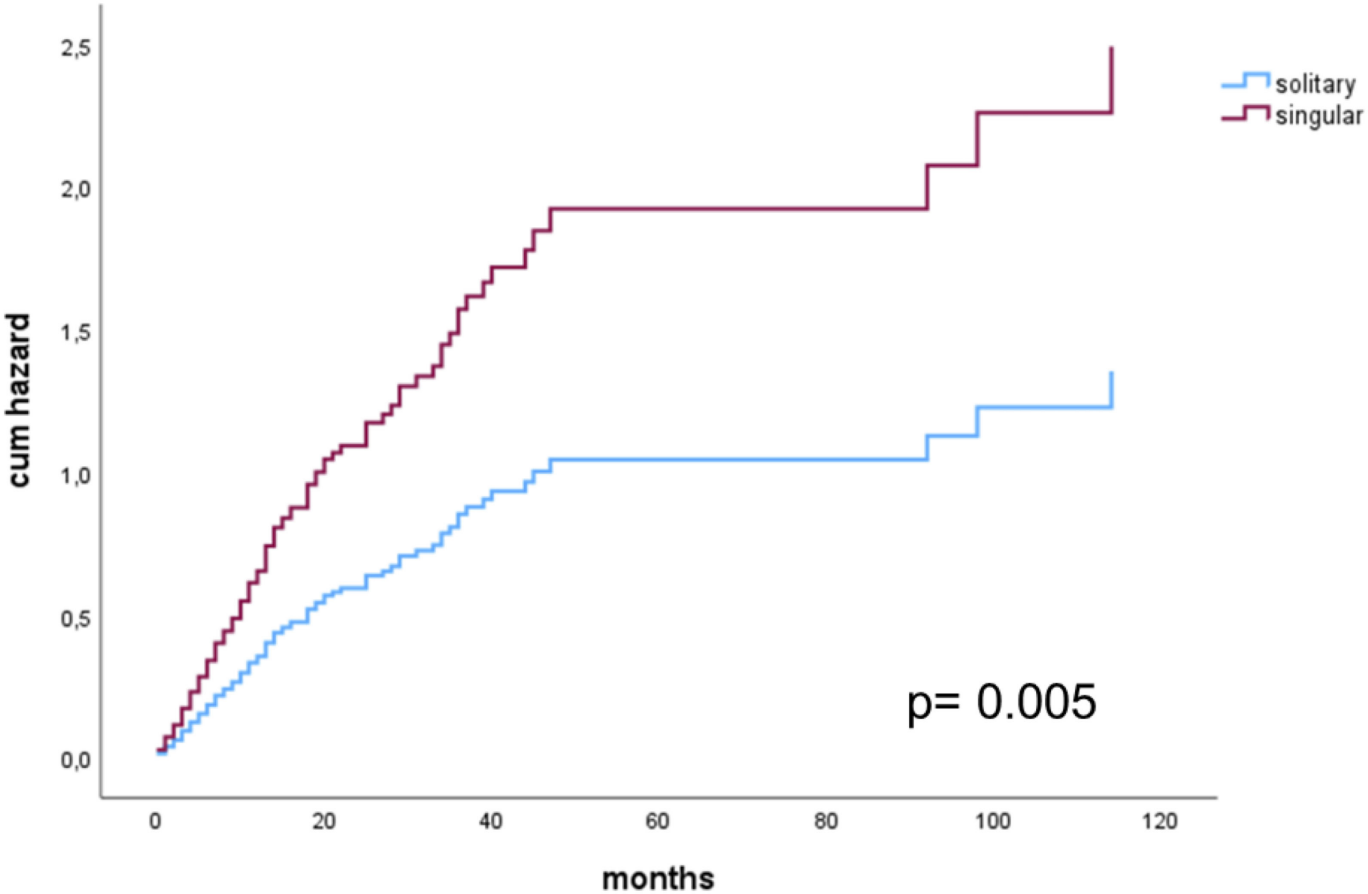
Cox proportional hazards model for singular versus solitary metastasis in patients with a complete resection.

Additionally, when only patients with a complete resection were considered, Cox regression multivariate analysis showed singular BM to be a significant risk factor for shorter OS (HR 1.840 (1.202-2.817), p=0.005). Sex, histology groups, post-op chemotherapy, post-op radiotherapy and singular/solitary BM were included as potential confounders (**Figure 4**).

### 3.4 Impact of postoperative radiotherapy

For 317 cases, detailed radiotherapy data were available. The remaining 23 patients received adjuvant therapy in external clinics, but detailed data were not available retrospectively.

233/317 (75%) patients underwent postoperative radiotherapy (RT). Whole brain radiotherapy (WBRT) was conducted in 75/317 (25%) patients. Stereotactic radiosurgery (SRS) was performed in 7/317 (2%) and hypofractionated Stereotactic Radiotherapy (HSRT) was conducted in 151/317 (48%) patients. Patients receiving postoperative RT had a significantly longer OS compared to patients without postoperative RT (14 months (IQR 7-42) vs. 4 months (IQR 1-17), p<0.001) with significant differences between the groups (p<0.001) and favorable survival rates especially in patients receiving SRS (OS median 15 months (3-42 months) and HSRT (15months (IQR 6-30)) compared to no RT (OS 4 months (IQR1-9) and WBRT (5 months (IQR1-16).

## 4 Discussion

The present study shows that patients with solitary BM have a significantly higher OS compared to patients with singular BM. Furthermore, adjuvant RT, especially SRS, was associated with significantly longer OS in all patients. We analyzed a very homogeneous cohort of patients regarding age, sex, tumor burden, KPSS and adjuvant treatment structure as shown in the baseline characteristics of the two groups (**Table 1**). This allows a valid comparison of the two study groups.

### 4.1 Survival analysis and impact of RTB

In our study there was no significant difference between patients with complete vs. incomplete resection regarding OS. Furthermore, smaller postoperative tumor burden had no significant influence on prolonged OS. This finding is in accordance with previous studies discussing the influence of gross total resection in patients with brain metastases (14). Rades et al. compared neurosurgical resection followed by WBRT vs. WBRT + SRS. The study proved WBRT + SRS to have a significantly better local control. There was no significant difference in OS (15). Another investigation compared SRS vs. surgical resection + WBRT. SRS alone was as effective as resection followed by WBRT for patients with a single BM (16). A systematic review by Qin et al. proved SRS and neurosurgical resection to be equally beneficial regarding OS for patients with single BM and lung cancers as primary tumor (17).

Therefore, the interdisciplinary decision whether to treat a single BM with radiotherapy/radiosurgery or surgical resection should be well-considered.

In our study, we found a clear trend that patients with solitary BM who underwent complete resection had a favorable prognosis compared to incomplete resection (OS: 21 vs. 8 months).

Although statistical significance was not achieved, there is a strong presumption that complete resection leads to an improved oncological outcome.

Conversely, among patients with singular BM, the progression of systemic metastases emerges as a pivotal prognostic factor, as that complete resection of the singular BM was not associated with a favorable OS. The progression of the systemic disease is an important factor, impacting the physical status of the patient and the prognosis.

According to Mintz et al. surgical resection should be considered for patients with good performance status, minimal or no evidence of extracranial disease, and a surgically accessible single BM amenable to complete excision with RTB=0 (18).

Patchell et al. emphasized that patients with surgically accessible lesions, either solitary BM or controlled systemic disease, and a life expectancy of ≥ 2 months may benefit from surgical resection. Patients with lower life expectancies or progressive diseases should rather be treated with palliation or radiotherapy (19).

### 4.2 Prognostic factors

Our study proved singular brain metastasis (i.e., the presence of extracranial metastases) to be a significant risk factor for shorter OS in patients with only one BM. This result is demonstrated in the analysis of all patients as well as in the subgroup analysis of patients with RTB=0 and consequently complete cytoreduction. The extent of the non-CNS disease and the progression of the systemic disease is an important factor impacting the physical status of the patient and the prognosis.

This finding is in accordance with previous studies (2, 20). The presence of further systemic metastases is indicative of advanced disease stage and therefore results in shorter OS in these patients.

### 4.3 Adjuvant therapy

Our study highlights that adjuvant radiotherapy is associated with significantly longer OS compared to no postoperative RT. This finding is in accordance with the recent literature (6).

This finding is of critical importance in order to select the optimal treatment approach for this cohort of patients. Since reduced postoperative tumor burden did not have a significant impact on prolonged OS, postoperative RT becomes even more imperative in patients with single BM.

Considering different types of radiotherapy, the present investigation shows significantly favorable survival rates especially in patients receiving SRS and HSRT compared to no RT or WBRT. Vlachos et al. compared SRS vs. WBRT after resection of solitary brain metastasis. They proved SRS to be as effective as WBRT in terms of OS. Furthermore, SRS was associated with superior quality of life (21). Kerschbaumer et al. also compared sector irradiation (SR) vs. WBRT after resection of singular BM in a prospective trial. Patients who underwent SR had longer OS and better neurocognitive function compared to WBRT. In terms of local control, more distant relapses occurred in the SR group (22).

The non-inferiority of SRS compared to WBRT could be demonstrated and confirmed in our present study. This is of utmost importance as SRS is able to improve the cognition and quality of life of patients in the long term with the same survival rates.

### 4.4 Study limitations

A major limitation of the present investigation is its retrospective character, as it restricted the data analysis to the presented parameters and introduces an unavoidable selection bias. A prospective study would be highly advantageous for further data, especially new histopathological findings, and new therapeutic options, that have dynamically expanded in the last years, to compare even more aspects. Therefore, this study cannot reflect the most recent therapy innovations. Nevertheless, the analyzed data, particularly the key results are based on reports that have been digitally documented, making them as reliable as in a prospective setup.

## 5 Conclusions

When complete intracranial tumor resection (RTB=0) is achieved, patients with solitary BM have a favorable outcome compared to patients with singular BM. In a multivariate Cox regression analysis, singular BM (compared to solitary BM) was confirmed as an independent risk factor. RTB did not have a significant impact on OS.

The highly advantageous impact of postoperative RT on OS was demonstrated and confirmed, especially in patients, receiving SRS or HSRT. Consequently, the decision whether to treat a patient with a single BM with microsurgical resection or radiotherapy alone should be weighed thoroughly. There is a strong presumption that complete resection leads to an improved oncological prognosis. Patients with solitary BM tend to benefit with a favorable outcome following complete resection. Hence, surgical resection should be considered as a treatment option for patients presenting with either no or minimal extracranial disease.

## Data availability statement

The raw data supporting the conclusions of this article will be made available by the authors, without undue reservation.

## Ethics statement

Ethical approval was not required for the studies on humans in accordance with the local legislation and institutional requirements because only commercially available established cell lines were used.

## Author contributions

LB: Writing – review & editing, Writing – original draft, Software, Methodology, Investigation, Data curation, Conceptualization. AA: Writing – review & editing, Writing – original draft, Investigation, Formal Analysis, Conceptualization. AKA: Writing – review & editing, Investigation. BW: Writing – review & editing, Supervision. DB: Writing – review & editing, Supervision. SC: Writing – review & editing, Supervision. HM: Writing – review & editing, Supervision. GS: Writing – review & editing. BM: Writing – review & editing, Supervision, Project administration. JG: Writing – review & editing, Supervision, Project administration, Conceptualization.

## References

[1] Soffietti R Abacioglu U Baumert B Combs SE Kinhult S Kros JM. Diagnosis and treatment of brain metastases from solid tumors: Guidelines from the European Association of neuro-oncology (EANO). Neuro Oncol. (2017) 19:162–74. doi: 10.1093/neuonc/now241 PMC5620494 28391295

[2] Schackert G Steinmetz A Meier U Sobottka SB. Surgical management of single and multiple brain metastases: Results of a retrospective study. Onkologie. (2001) 24:246–55. doi: 10.1159/000055087 11455217

[3] Medikonda R Jackson CM Feghali J Lim M. The effects of postoperative neurological deficits on survival in patients with single brain metastasis. Oper Neurosurg. (2020) 19:628–34. doi: 10.1093/ons/opaa224 32717025

[4] Lagerwaard FJ Levendag PC Nowak PJCM Eijkenboom WMH Hanssens PEJ Schmitz PIM. Identification of prognostic factors in patients with brain metastases: A review of 1292 patients. Int J Radiat Oncol Biol Phys. (1999) 43:795–803. doi: 10.1016/S0360-3016(98)00442-8 10098435

[5] Hall WA Djalilian HR Nussbaum ES Cho KH. Long-term survival with metastatic cancer to the brain. Med Oncol. (2000) 17:279–86. doi: 10.1007/BF02782192 11114706

[6] Mahajan A Ahmed S McAleer MF Weinberg JS Li J Brown P. Post-operative stereotactic radiosurgery versus observation for completely resected brain metastases: a single-center, randomized, controlled, phase 3 trial. Lancet Oncol. (2017) 18:1040–8. doi: 10.1016/S1470-2045(17)30414-X PMC5560102 28687375

[7] Aftahy AK Barz M Lange N Baumgart L Thunstedt C Eller MA. The impact of postoperative tumor burden on patients with brain metastases. Front Oncol. (2022) 12:869764. doi: 10.3389/fonc.2022.869764 35600394 PMC9114705

[8] Stummer W Pichlmeier U Meinel T Wiestler OD Zanella F Reulen H-J. Fluorescence-guided surgery with 5-aminolevulinic acid for resection of Malignant glioma: a randomized controlled multicenter phase III trial. Lancet Oncol. (2006) 7:392–401. doi: 10.1016/S1470-2045(06)70665-9 16648043

[9] Stummer W Reulen H-J Meinel T Pichlmeier U Schumacher W Tonn J-C. Extent of resection and survival in glioblastoma multiforme: identification of and adjustment for bias. Neurosurgery. (2008) 62. doi: 10.1227/01.neu.0000317304.31579.17 18425006

[10] Olesrud IC Schulz MK Marcovic L Kristensen BW Pedersen CB Kristiansen C. Early postoperative MRI after resection of brain metastases—complete tumor resection associated with prolonged survival. Acta Neurochir (Wien). (2019) 161:555–65. doi: 10.1007/s00701-019-03829-0 30756241

[11] Orringer D Lau D Khatri S Zamora-Berridi GJ Zhang K Wu C. Extent of resection in patients with glioblastoma: limiting factors, perception of resectability, and effect on survival: Clinical article. J Neurosurg JNS. (2012) 117:851–9. doi: 10.3171/2012.8.JNS12234 22978537

[12] Senft C Bink A Franz K Vatter H Gasser T Seifert V. Intraoperative MRI guidance and extent of resection in glioma surgery: a randomized, controlled trial. Lancet Oncol. (2011) 12:997–1003. doi: 10.1016/S1470-2045(11)70196-6 21868284

[13] Kiesel B Thomé CM Weiss T Jakola AS Darlix A Pellerino A. Perioperative imaging in patients treated with resection of brain metastases: a survey by the European Association of Neuro-Oncology (EANO) Youngsters committee. BMC Cancer. (2020) 20:410. doi: 10.1186/s12885-020-06897-z 32398144 PMC7216695

[14] Jünger ST Pennig L Schödel P Goldbrunner R Friker L Kocher M. The debatable benefit of gross-total resection of brain metastases in a comprehensive treatment setting. Cancers (Basel). (2021) 13. doi: 10.3390/cancers13061435 PMC8004079 33801110

[15] Rades D Veninga T Hornung D Wittkugel O Schild SE Gliemroth J. Single brain metastasis: whole-brain irradiation plus either radiosurgery or neurosurgical resection. Cancer. (2012) 118:1138–44. doi: 10.1002/cncr.26379 21761403

[16] Rades D Bohlen G Pluemer A Veninga T Hanssens P Dunst J. Stereotactic radiosurgery alone versus resection plus whole-brain radiotherapy for 1 or 2 brain metastases in recursive partitioning analysis class 1 and 2 patients. Cancer. (2007) 109:2515–21. doi: 10.1002/cncr.22729 17487853

[17] Qin H Wang C Jiang Y Zhang X Zhang Y Ruan Z. Patients with single brain metastasis from non-small cell lung cancer equally benefit from stereotactic radiosurgery and surgery: A systematic review. Med Sci Monit. (2015) 21:144–52. doi: 10.12659/MSM.892405 PMC4299005 25579245

[18] Mintz A Perry J Spithoff K Chambers A Laperriere N. Management of single brain metastasis: A practice guideline. Curr Oncol. (2007) 14:131–43. doi: 10.3747/co.2007.129 PMC1948870 17710205

[19] Patchell RA Tibbs PA Regine WF Dempsey RJ Mohiuddin M Kryscio RJ. Postoperative radiotherapy in the treatment of single metastases to the brain: A randomized trial. J Am Med Assoc. (1998) 280:1485–9. doi: 10.1001/jama.280.17.1485 9809728

[20] Noordijk EM Vecht CJ Haaxma-Reiche H Padberg GW Voormolen JHC Hoekstra FH. The choice of treatment of single brain metastasis should be based on extracranial tumor activity and age. Int J Radiat Oncol Biol Phys. (1994) 29:711–7. doi: 10.1016/0360-3016(94)90558-4 8040016

[21] Vlachos N Lampros MG Filis P Voulgaris S Alexiou GA. Stereotactic radiosurgery versus whole-brain radiotherapy after resection of solitary brain metastasis: A systematic review and meta-analysis. World Neurosurg X. (2023) 18:100170. doi: 10.1016/j.wnsx.2023.100170 36825221 PMC9942116

[22] Kerschbaumer J Pinggera D Holzner B Delazer M Bodner T Karner E. Sector irradiation vs. Whole brain irradiation after resection of singular brain metastasis—A prospective randomized monocentric trial. Front Oncol. (2020) 10:591884. doi: 10.3389/fonc.2020.591884 33330076 PMC7732624

